# A unified and flexible modelling framework for the analysis of malaria serology data

**DOI:** 10.1017/S0950268821000753

**Published:** 2021-04-12

**Authors:** Irene Kyomuhangi, Emanuele Giorgi

**Affiliations:** CHICAS, Lancaster Medical School, Lancaster University, Lancaster, UK

## Abstract

Serology data are an increasingly important tool in malaria surveillance, especially in low transmission settings where the estimation of parasite-based indicators is often problematic. Existing methods rely on the use of thresholds to identify seropositive individuals and estimate transmission intensity, while making assumptions about the temporal dynamics of malaria transmission that are rarely questioned. Here, we present a novel threshold-free approach for the analysis of malaria serology data which avoids dichotomization of continuous antibody measurements and allows us to model changes in the antibody distribution across age in a more flexible way. The proposed unified mechanistic model combines the properties of reversible catalytic and antibody acquisition models, and allows for temporally varying boosting and seroconversion rates. Additionally, as an alternative to the unified mechanistic model, we also propose an empirical approach to analysis where modelling of the age-dependency is informed by the data rather than biological assumptions. Using serology data from Western Kenya, we demonstrate both the usefulness and limitations of the novel modelling framework.

## Introduction

Despite the significant progress made in the control of malaria worldwide, this still remains a significant public health threat in many countries, particularly in Sub-Saharan Africa [1]. Even with the decline of malaria prevalence in endemic countries [2], there are still challenges that require robust mechanisms for monitoring malaria transmission and evaluation of elimination efforts [1].

Classical methods of estimating malaria risk rely on the detection of the *Plasmodium* parasite in humans and mosquito populations. *Plasmodium falciparum (Pf)* is the most prevalent malaria parasite in Africa, while *Plasmodium vivax (Pv)* dominates in the Americas and South East Asia [1]. Parasite prevalence is determined by the proportion of infected individuals at the time of data collection [3, 4], while the entomological inoculation rate (EIR) is the rate at which individuals are bitten by infectious mosquitoes [5]. Both of these measures may vary over time due to the joint effect of several environmental factors, and the precision with which they can be estimated is often low, particularly in low transmission settings [3, 4]. Additionally, the collection of entomological data is labour-intensive, expensive and excludes the recruitment of children, due to ethical considerations [6–8].

Several studies have shown the utility of serological markers as a viable alternative for estimating transmission intensity. Because of the persistence of antibodies, serological markers (1) provide information on cumulative exposure to the pathogen over time, (2) smooth out the effect of seasonality in transmission, and (3) allow estimation of transmission intensity with more feasible sample sizes even in low transmission settings [3, 8–10].

Antibody responses to blood-stage malaria parasites provide protection against clinical disease, however this response does not confer sterile immunity, therefore individuals remain susceptible to repeated infections [11, 12]. In malaria endemic settings, antibody levels generally increase as individuals become older, are boosted by repeated infection and decay in the absence of re-infection [4, 13]. Using existing knowledge on the dynamics of transmission, malaria serology models aim to derive a measure of transmission which can be used to monitor trends in endemic areas over time.

The most commonly used approach to estimate malaria transmission intensity is based on the classification of individuals as seronegative and seropositive which is then used as the input of a reversible catalytic model (RCM), to estimate the seroconversion rate, which quantifies the rate at which individuals convert from seronegative to seropositive [4, 8, 9]. Assuming latent seronegative and seropositive distributions in the sample, mixture models fitted to the antibody distribution are used in order to identify optimal thresholds for the classification of individuals into seropositives and seronegatives [4, 14]. The major drawback of this approach is that it can generate biased estimates of transmission intensity as a result of the misclassification, especially among inconclusive cases whose probabilities of belonging to either group are close to 50% [15, 16]. Bollaerts *et al*. [15] and Hens *et al*. [16] propose a ‘direct’ method of estimating seroprevalence from continuous antibody measurement using an underlying mixture model, which avoids the use of thresholds and thus the bias arising from the misclassification of individuals. In those publications, the direct method is applied to Salmonella and Varicella-Zoster virus antibody data. This approach has not been applied to analyse malaria serology data and, in this paper, we propose a modelling framework that is inspired by Hens *et al*. [16].

In addition to the seroconversion rate, boosting rates, i.e. the rate at which antibody levels are acquired, can also be used as a marker for transmission intensity [4, 17, 18]. Antibody acquisition models (AAMs) have been developed as an alternative approach to RCMs, and do not involve the use of thresholds but instead rely on the full antibody measurements in order to estimate boosting rates. However, in the context of malaria serology, current formulations of the AAM assume that the antibody measurements follow a log-Gaussian distribution, clearly an invalid assumption in the case of a bi-modal distribution arising from the mixing of the seropositive and seronegative populations [17].

RCMs and AAMs that have been applied to the analysis of malaria serology data make strong assumptions on the temporal dynamics of transmission, which are generally restricted to the following patterns: constant transmission, a sharp stepwise drop in transmission and a linear drop in transmission [4, 17–19]. The validity of these assumptions is often questionable, and more flexible functional forms for the variation of transmission over time have not been considered in the context of malaria serology.

In this paper, we develop a unified mechanistic model for the analysis of malaria serology data which combines the properties of mixture models, RCMs and AAMs in order to reliably estimate malaria transmission intensity. We also show that the additional flexibility brought by this novel model allows a better description of temporal dynamics of malaria transmission. In addition to this, we present an alternative empirical approach to account for the age-dependency of the antibody distributions and use this approach to validate the unified mechanistic model.

The structure of the paper is as follows. Section ‘Existing models’ provides an overview of current models for malaria serology analysis. Section ‘A unified mechanistic model for the analysis of malaria serology data’ introduces a unified mechanistic model and outlines an alternative empirical approach that can be used to analyse malaria serology data. In section ‘Analysis of malaria serology data from Western Kenya’, we apply this new framework to cross-sectional antibody data from Western Kenya, and section ‘Discussion’ is a discussion of the results. Finally, section ‘Conclusion’ provides a summary and conclusion.

## Existing models

### Mixture models

In the context of malaria and other infectious diseases, mixture models are developed under the assumption that the population of interest is indeed a mixture of latent seropositive and seronegative populations [4, 20]. More formally, let *Y*_*i*_ denote the log-transformed antibody measurement for the *i*-th individual. Let *S*^+^ and *S*^−^ be a shorthand notation for ‘seropositive’ and ‘seronegative’ classifications, respectively. Assuming independent and identically distributed realisations for a sample of *n* individuals, we write the density function of *Y*_*i*_ as1
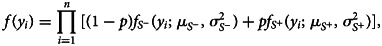
where 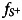 is a univariate log-Gaussian distribution with mean 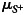 and variance 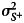 for the *S*^+^ population, and analogously for *S*^−^; finally, *p*is the probability of being *S*^+^.

Let *C*_*i*_ and 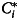 denote the random variables representing classification based on the mixture model and true classification of the *i*-th individual, respectively. One approach is to define a seropositivity threshold, usually 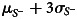, above which *C*_*i*_ is *S*^+^, and *S*^−^ if below [4, 15, 16, 19, 21]. An alternative, more elaborate, approach is to first calculate the probability of belonging to group 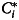, conditional on the antibody measurement *Y*_*i*_ = *y*_*i*_, i.e.2
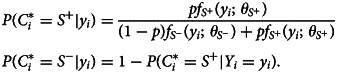


Based on two probability thresholds, *c*^−^ and *c*^+^, the classification *C*_*i*_ is3
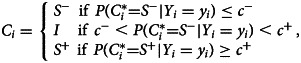
where *I* is an additional classification label introduced to denote inconclusive cases. In serology analysis, a common approach is to exclude these cases, depending on the type of disease, and report the proportion of inconclusive cases [15, 16, 22].

In malaria serology, most studies favour the first threshold-based approach that does not introduce the classification for inconclusive cases [17–19, 23–25]. This is likely due to the nature of antibody responses to malaria infections which result in a large proportion of ‘inconclusive’ cases, as reported by Sepúlveda *et al*. [4].

However, both of these threshold-based approaches are prone to misclassification, which can create bias in estimating epidemiological parameters [4, 15, 16]. Furthermore, current applications of mixture models in malaria serology analysis do not take into account the age-dependence of antibody levels, and assume that the mixing of *S*^+^ and *S*^−^ is the same across all ages, which may further exacerbate the issue of misclassification.

The two component mixture Gaussian models also do not account for antibody boosting upon re-exposure to malaria parasites. Sepúlveda *et al*. [4] present an extension to the traditional mixture model where more components are added in order to account for this boosting effect. These components can be interpreted as varying degrees of malaria exposure; unexposed, once exposed, twice exposed, etc. Assuming a known number of components, say *K*, the sampling distribution is given by4
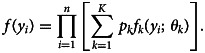


The number of components *K* is then treated as an additional parameter to estimate using the profile likelihood. However, the interpretation of the components of the model is problematic due to ambiguity about classification rules, particularly when component means are close together. This approach also further compounds the problem of inconclusive cases as they occur across multiple components.

**Fig. 1.**
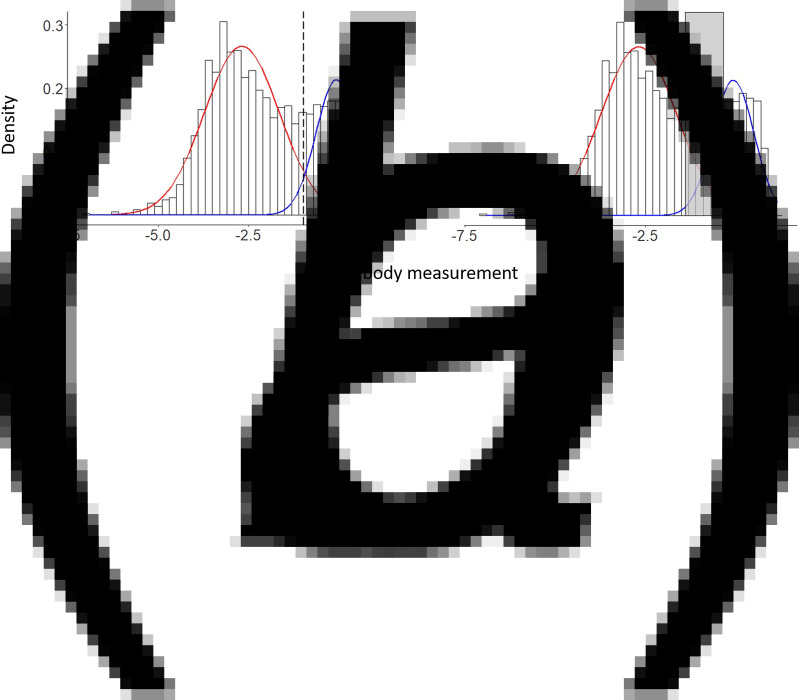
An illustration of the mixture model showing the bi-modal distributions for the *S*^−^ (red) and *S*^+^ (blue) populations. The dotted line in (a) shows the seropositivity threshold 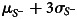, above which individuals are classified as *S*^+^. The grey rectangle in (b) shows the inconclusive cases as defined by equation (3). In this case, the probability thresholds *c*^−^ and *c*^+^ have been set to 90%. Individuals below this grey region are classified as *S*^−^, while individuals above this region are classified as *S*^+^. These data are taken from the *Pf* AMA1 analysis in section ‘Analysis of malaria serology data from Western Kenya’.

### Reversible catalytic models

**Fig. 2.**
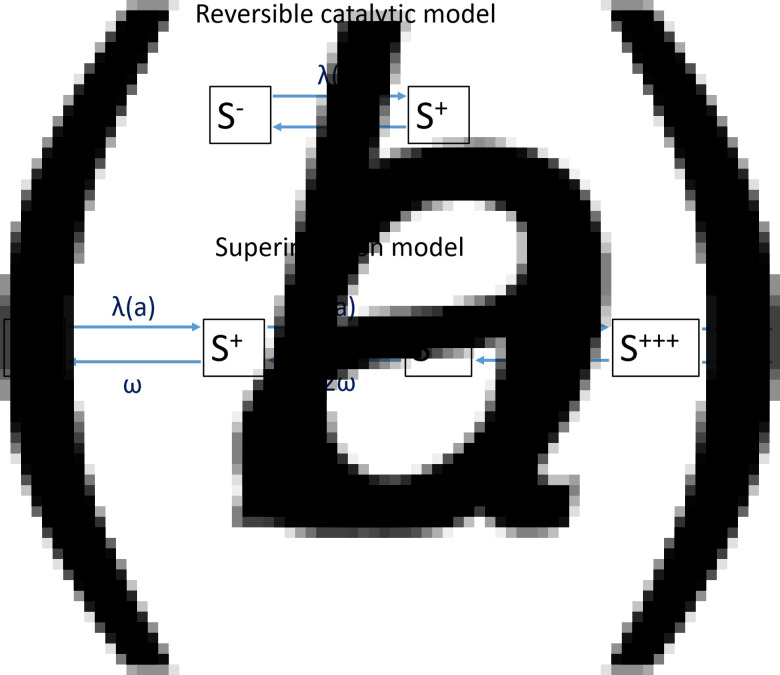
(a) This figure is a representation of the reversible catalytic model (RCM) where individuals transition between seronegative (*S*^−^) and seropositive (*S*^+^) states through the SCR, *λ*(*a*)and the SRR, *ω*. (b) This figure is a representation of the superinfection model (SIM) where individuals can have their antibodies ‘boosted’ through increasing seropositive (*S*^+…^) states depending on the cumulative exposure to malaria parasites.

Following the dichotomisation of the continuous antibody measurements through the application of a mixture model, the resulting *S*^+^ and *S*^−^ outcomes are modelled using an RCM. A common assumption of the RCM is that individuals are born *S*^−^ and, after becoming *S*^+^ upon exposure to malaria, can revert to *S*^−^ in the absence of exposure. This mechanistic approach is illustrated in Figure 2a. Since antibody data are assumed to represent the cumulative exposure of individuals during their lifespan, the age of individual prior to the sample collection is used as a proxy for historical time.

Let *λ*(*a*) denote the seroconversion rate for an individual at age *a* and *ω* the seroreversion rate. According to the RCM, the temporal dynamics that regulate the proportion of *S*^+^ individuals of age *a*, hence *p*(*a*), are expressed by the following differential equation5
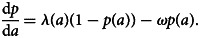


In the above equation, *λ*(*a*) is a measure of the underlying transmission intensity which is associated with the gold standard indicator of transmission, the EIR [8], while *ω* is typically fixed and assumed to be constant [4]. However, some authors Bosomprah [19] and Akpogheneta *et al*. [26] suggest that *ω* may be age-dependent. Sepúlveda *et al*. [4] argue that the malaria serology data often carry little information in the estimation of *ω*, a problem which will persist also in our novel modelling framework. Hence, throughout this paper, we shall make the working assumption of a constant *ω*. Note that the reciprocals of *λ* and *ω* estimates, i.e. 1/*λ* and 1/*ω*, indicate the estimated number of years within which seroconversion and seroreversion would occur, respectively.

Three transmission profiles have so far been proposed to model the seroconversion rate *λ*(*a*). The simplest assumes a constant transmission, hence *λ*(*a*) = *λ* for all *a*. In this case, the differential equation in (5) gives the following solution6
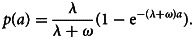


In the equation above, the proportion of *S*^+^ at older ages reaches a maximum value of about *λ*/(*λ* + *ω*). In other words, in a cohort of an initially malaria-naive population, *p*(*a*) will ultimately reach a plateau at which the number of individuals seroconverting is the same as the number of individuals seroreverting [4, 8]. However, these assumptions may be too stringent as they ignore changes in transmission that may be due, for example, to the introduction of control interventions [4, 20, 21].

To tackle this issue, one approach is to assume a transmission profile with a sharp drop in transmission at the time of intervention. In this model, two transmission rates are estimated: *λ*_1_ and *λ*_2_ which represent the transmission rates before and after the drop, respectively. An alternative approach to account for control interventions is to assume a linear reduction in the seroconversion rate *λ*(*a*), rather than a step-change as we have just illustrated. However, in this case, the differential equation in (5) cannot be solved analytically and numerical procedures must instead be used.

In the study by Yman *et al*. [17], the two transmission profiles that do not assume a constant *λ*(*a*) provide a better fit to the data. However, assumption of a step-change or linear drop in *λ*(*a*) may be inappropriate in the presence of major or prolonged malaria outbreaks within the historical time-frame considered. In general, the validity of any of these profiles is dependent on a variety of factors, including intervention history, climate and vector characteristics. More recently, Varela *et al*. [27] propose a model where the number of times that *λ* changed in the past, which is also estimated from the data.

Where seropositivity is defined using the traditional two-component Gaussian mixture model, there is still the issue of how to account for antibody boosting due to repeated exposure to malaria parasites. Bosomprah [19] suggests an extension to the RCM, which involves creating more seropositive classes in a superinfection model (SIM), similar to the multi-component mixture model described by Sepúlveda *et al*. [4]. In this framework, a seronegative individual can transition to the first seropositive class, *S*^+^, upon first exposure, and subsequently to a higher seropositive class *S*^++^ upon re-exposure, and so on, as illustrated in Figure 2b. The SIM also faces challenges with interpretation of results where initial exposure and boosting between the multiple seropositive classes may be conflated [3, 4].

### Antibody acquisition models

An alternative modelling approach to estimate transmission intensity is to use AAMs [17, 18]. Unlike RCMs, AMMs use the full antibody measurements without requiring any dichotomisation of the data. More specifically, AAMs are used to estimate the boosting rate, i.e. the rate at which antibodies are acquired, a marker for transmission intensity [4, 17, 18, 28]. Let *μ*(*a*) denote the average antibody level in the general population of individuals of age *a*. Assuming that following exposure to parasites, *μ*(*a*) is boosted at a rate *γ*(*a*) and assuming a constant decay rate *r*, we can express this mechanism through the following differential equation7
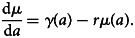


We can then use the above equation to infer changes in average antibody levels as a function of age *a*. Finally, in order to fit (7) using likelihood-based methods of inference, the antibody levels of individuals at age *a* are assumed to follow a log-Gaussian distribution with mean *μ*(*a*) and variance *σ*^2^ [4, 17, 18].

Similar to the way the way seroconversion rates have been modelled in RCMs (section ‘Reversible catalytic models’), previous studies have considered three transmission profiles for the specification of *γ*(*a*). The simplest approach assumes that *γ*(*a*) = *γ* is constant which leads to the following solution of (7)8
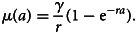


Similarly to RCMs, extensions of the AAM assumes either a step-change or linear reduction in the boosting rate *γ*; see Sepúlveda *et al*. [4], Yman *et al*. [17] and Weber *et al*. [18] for more details.

Direct comparison of *γ* and *λ* from the AAM and RCM, respectively, may not be possible as these estimate different serological indicators. However, Yman *et al*. [17] find that the AAMs provide a more consistent fit to age-dependent antibody data compared to RCM fit to age-dependent seroprevalence data. Additionally, AAMs provide better precision in parameter estimation and appear to be more robust to sample size reduction. It has been found that AMMs often provide a good fit to serological data in high to moderate transmission settings, where a large proportion of individuals may be seropositive [17], or where an antigen is highly immunogenic, leading to high seropositivity to its antibody in the population [18].

## A unified mechanistic model for the analysis of malaria serology data

In this section, we develop a statistical modelling framework which extends the standard mixture model outlined in section ‘Mixture models’ to incorporate both the RCM and AAM dynamics and provides a more flexible approach to model time changes in the seroconversion rate and boosting rate. In this unified framework, the mixing probabilities – i.e. probability of belonging to the *S*^+^ and *S*^−^ populations – are modelled based on the RCM, while the means of the two latent *S*^+^ and *S*^−^ distributions are informed by AAM dynamics.

To avoid the need of solving complex differential equations, we re-express (5) with a discrete-time difference equation, i.e.
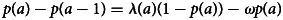
or, equivalently,
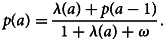


Assuming that *λ*(0) = 0, and by iteratively applying the above expression, we then obtain9
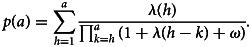


This allows us to specify any function for *λ*(*a*) without being constrained to three options described in section ‘Reversible catalytic models’. The above describes the proportion of *S*^+^ individuals who are aged *a*, *p*(*a*), as a weighted sum of transmission intensities occurring in all the years since birth, *λ*(*h*), with weights decreasing exponentially as we move further back in time from the time of data collection.

We apply this same idea to the AAM, allowing for temporally varying *γ*(*a*). More specifically, by using a discrete-time dynamic we re-write (7) as
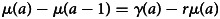
or, equivalently,
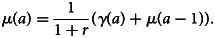


By applying the above expression iteratively and assuming that *γ*(0) = 0, we obtain that10
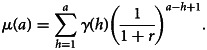


Similar to the interpretation of (9), in this expression, the mean antibody level at age *a*, *μ*(*a*), is given by weighted sum of all the boosting rates since birth, *γ*(*h*), and the weights given are exponentially decaying. The assumptions of *λ*(0) = 0 and *γ*(0) = 0 may not be strictly valid, however, this is a pragmatic choice since the true boosting and seroconversion rates at birth are not known but are expected to be close to zero on account of underdeveloped immune responses to malaria in infants who rely on maternal antibodies up to 9 months after birth [28–30].

To model the temporal changes in *λ*(*h*) and *γ*(*h*), in the absence of a detailed information on intervention history, a pragmatic approach is to use a log-linear regression in the years before the time of data collection, which is expressed as11
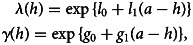
where *h* corresponds to a given age of an individual before the time of collection and, thus, *a* − *h*is the years before the time of data collection. Finally, *l*_0_, *l*_1_, *g*_0_ and *g*_1_ are regression parameters to estimate (Fig. S1 of the Supplementary material further illustrates the mechanism of this approach).

**Fig. 3.**
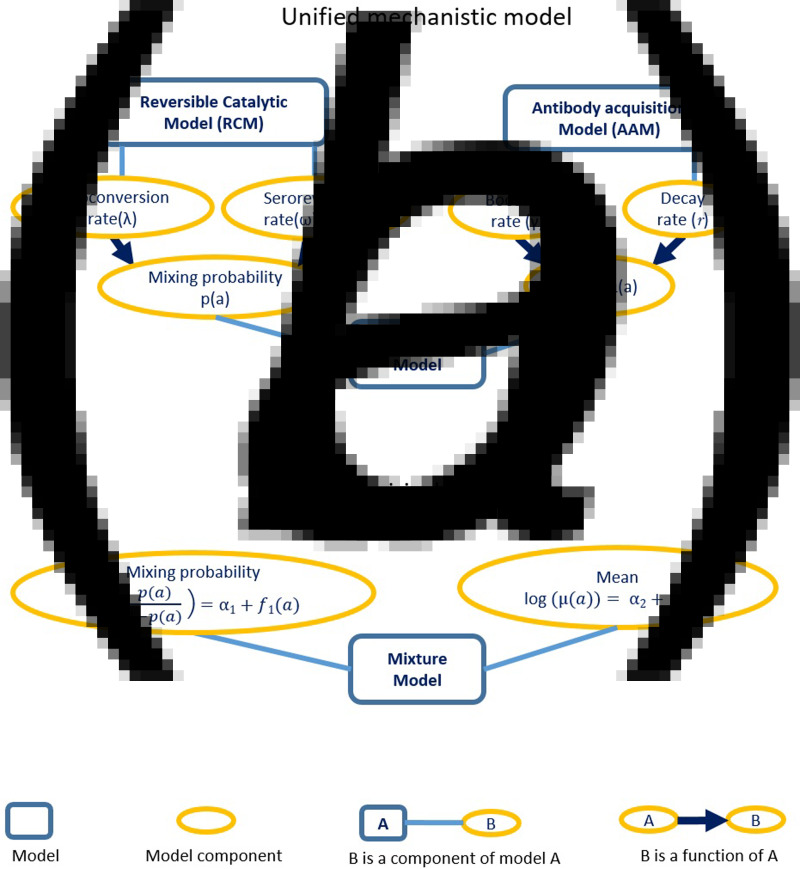
(a) This figure is a representation of the unified mechanistic model, showing how the reversible catalytic model and antibody acquisition model are incorporated into the mixture model for antibody data. (b) This figure is a representation of the empirical model used to model age-dependence in the mixing probabilities and mean antibody level.

Assuming *μ*(*a*_*i*_) in (10) to be the mean level of antibodies in the *S*^−^ population, the density function of the resulting mixture model using the ‘direct’ approach is12
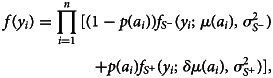
where *δ* > 1 is a multiplicative factor accounting for the higher mean levels of antibodies in the *S*^+^ population. In the ‘direct’ approach, we utilise the underlying structure of the mixture distribution in order to estimate transmission parameters in the unified mechanistic model, thus avoiding dichotomisation of the antibody measurements while accounting for age dependency of the mean and probabilities of the mixture. The resulting structure of the unified mechanistic model is summarised in Figure 3(a).

When analysing cross-sectional data, estimation of the model in (12) can be problematic because of the large number of parameters to estimate. In the absence of a large amount of data, the approach we follow in this paper is to consider two models, one assuming a time-varying seroconversion rate and a constant boosting rate, and a second where the reverse is assumed. Comparison between the two models is then carried out based on a goodness-of-fit index, such as the Akaike Information Criterion (AIC).

Another simplification that we introduce in the maximisation of the likelihood function is to fix the seroreversion rate *ω*. In practice, we found that using numerical optimisation with a continuous *ω* was unstable as a result of a very flat likelihood surface.

### Alternative empirical approaches to model age-dependency

When the interest is in describing the effect of age on the distribution of antibody data, an empirical, rather than mechanistic approach, may provide a better statistical solution. Additionally, the empirical approach outlined in this section can be used to validate the unified mechanistic model by assessing the discrepancy between the age distributions generated by the two modelling approaches.

To this end, we modify the framework introduced in the previous section by replacing the modelling of mixing probability based on RCMs, and the mean level of antibodies based on AAMs, with their empirical counterparts. More specifically, we model the age-dependency in *λ*(*a*) and *p*(*a*) using a log-linear and logit-linear regression as13
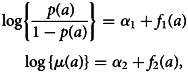
where *f*_1_(*a*) and *f*_2_(*a*) are functions that can be specified with the aid of simple graphical tools, such as scatter plots. The resulting structure of the empirical model is summarised in Figure 3b, and we give examples of this in the application of section ‘Analysis of malaria serology data from Western Kenya’.

## Analysis of malaria serology data from Western Kenya

We analyse data collected from a cross-sectional survey conducted in Rachuonyo South District, in the western Kenyan highlands, in 2011. At the time, malaria transmission in Rachuonyo South was described as generally low but highly heterogeneous, with an average of 14.8% malaria prevalence [31]. Transmission was characterised as seasonal, following peaks in rainfall, typically between March–June and October–November [31, 32].

Most malaria was attributed to *Pf*, with predominant vector species being *Anopheles gambiae s.s.*, *A. arabiensis* and *A. funestus* [33, 34]. Malaria control interventions at the time included the distribution of long-lasting insecticide-treated nets which had been ongoing for many years, and indoor residual spraying which started in 2009 [34]. Further details of the study design and data collection can be found in Bousema *et al*. [31, 34]

In the study, finger prick blood was collected from all participants on filter paper and used to detect total immunoglobulin G antibodies against the blood-stage *Pf* antigen apical membrane antigen 1 (*Pf* AMA1) using the enzyme-linked immunosorbent assay. Optical density (OD) values were obtained for this antigen and are the outcome that we model in this analysis, which we restrict to individuals between 1 and 16 years of age. Children under 1 year old are excluded from the analysis due to the effect of maternal antibodies, which are present at birth, and are believed to wane between 6 and 9 months [9, 17, 35]. The upper age range of 16 years is selected to exclude older individuals whose antibody levels may exhibit a noisier distribution and thus hinder the ability of the model to detect changes in transmission in the recent past from the time of data collection [17].

**Fig. 4.**
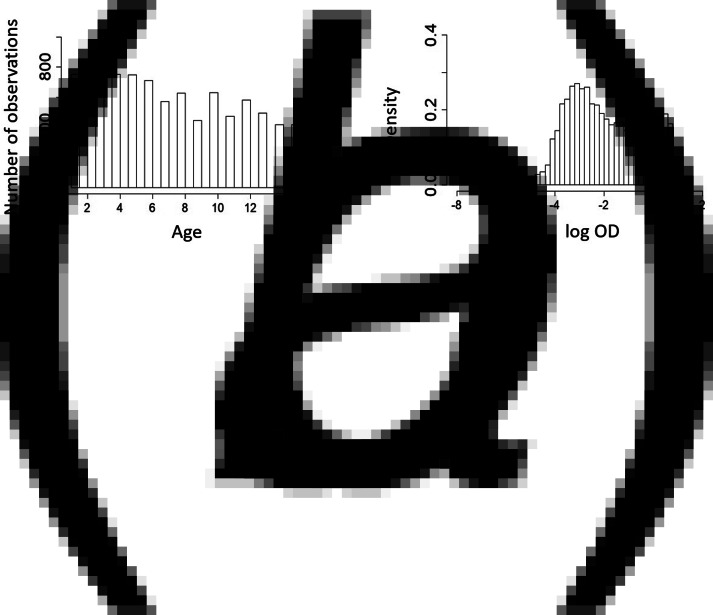
Descriptive plots of the age distribution (a) and the log OD distribution (b) of individuals aged 1–16, who are included in the *Pf* AMA1 antibody analysis.

The data-set consists of *n* = 9549 children. Figure 4 shows the age and OD distributions of the individuals included in the analyses.

We fit both unified mechanistic and empirical models to the *Pf* AMA1 antibody data using the maximum likelihood method of estimation. To obtain 95% confidence intervals (CIs) for the model parameters estimates, we use parametric bootstrap. In this procedure, parameter estimates from the respective models are used to generate 1000 replicate datasets. For each of the datasets, we refit the model and re-extract the parameter estimates in order to construct the bootstrap distribution, and therefore the CIs. We also account for the truncated nature of the antibody distributions, due to the exclusion of individuals under age 1 and over age 16, by using truncated log-Gaussian distributions. The upper limit of the truncation is estimated for each age group as the maximum observed value of OD.

Based on the comparison between the AIC values (see Table 2 in the Supplementary material), preliminary analysis of the *Pf* AMA1 data shows that a unified mechanistic model that assumes a time-varying seroconversion rate *λ*(*a*) and a constant boosting rate *γ* provides a better fit to the data than a model where the reverse assumptions is made (i.e. constant *λ* and time varying *γ*(*a*)). We let *ω* take three values, namely 0.01, 0.5 and 1, hence assuming that seroreversion events among individuals would occur between 1 and 100 years [8, 9, 26]. In what follows, we present results for the best performing value for *ω*, i.e. *ω* = 0.01.

To summarise, the unified mechanistic model parameters to estimate via maximum likelihood are the following: *l*_0_and *l*_1_ which are related to the seroconversion rate *λ* as described by (9) and (11); boosting rate *γ* and decay rate *r* from (10); and the mixture distribution parameters *δ*, 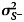 and 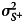 from (12).

**Fig. 5.**
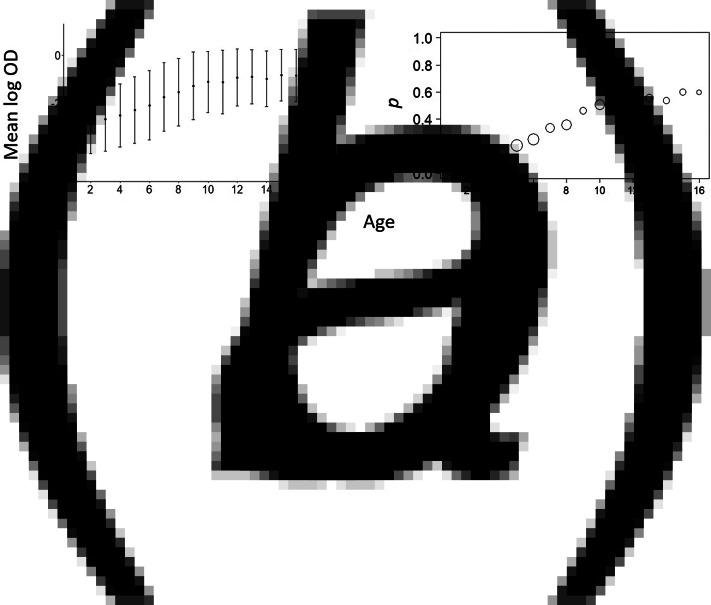
Exploratory analysis of the Rachuonyo South District *Pf* AMA1 antibody data. (a) This figure shows the geometric mean OD across age while (b) shows the proportions of *S*^+^ individuals, *p*, as defined by (1), using the seropositivity threshold (i.e. 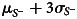). The circle sizes in (b) are proportional to the sample size in each age group.

For the empirical model, *μ*(*a*) and the mixing probability are modelled according to (13), and are informed by Figure 5. We apply a linear spline with a knot at age 10, based on the empirical trend for *μ*(*a*) observed in Figure 5a, to give14
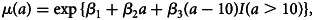
where *I*(*a* > 10) is an indicator function that takes value 1 if *a* > 10, and 0 otherwise. Based on Figure 5b, we introduce the log-transformed age as a logit-linear predictor for *p*(*a*), such that15
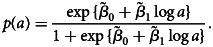

Thus, the model parameters to estimate for the empirical model are: the regression coefficients *β*_1_, *β*_2_ and *β*_3_ in (14), and 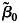 and 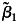 in (15); and, as in the unified model, *δ*, 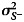 and 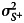.

**Fig. 6.**
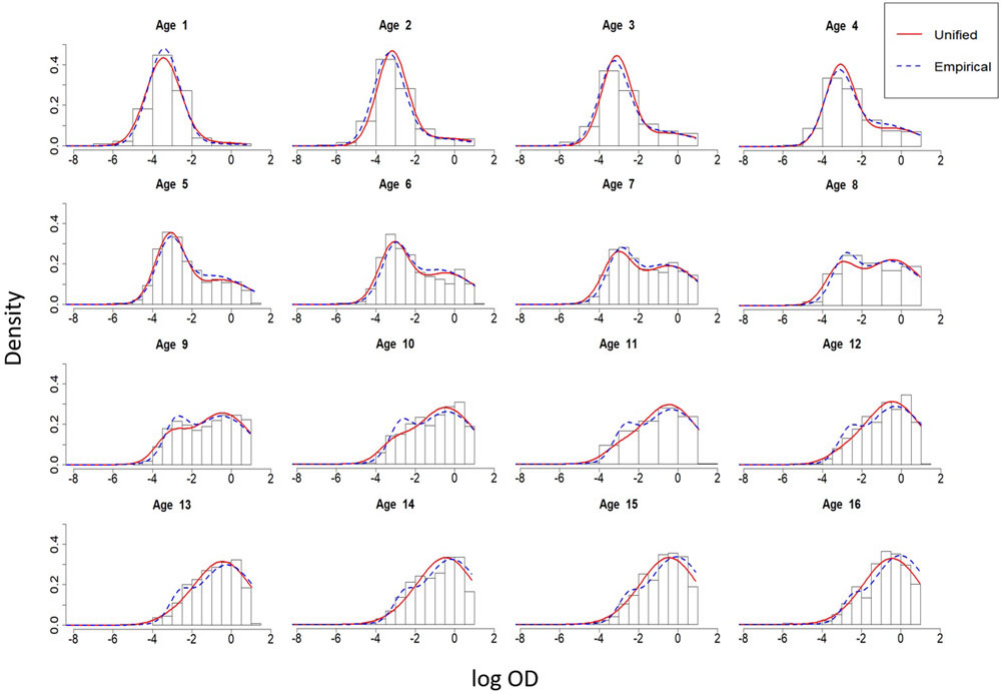
Age-dependent mixture distributions of *Pf* AMA1 antibodies for individuals 1–16 years of age in Rachuonyo South District. The red line indicates distributions derived from the unified mechanistic model, while the blue dotted line indicates distributions derived from the alternative empirical model.

Results of this analysis indicate strong evidence of age-dependency for the mixing probabilities of *Pf* AMA1. Figure 6 shows a bi-modal antibody distribution between ages 5 and 10, which is less evident in younger and older individuals. Both the empirical and mechanistic models are able to capture the increase in the means of antibodies for the *S*^+^ and *S*^−^ distributions, with younger children having generally lower antibody levels than older individuals.

By comparing the fitted density functions of mixture distributions between the mechanistic and empirical models for *Pf* AMA1 (Fig. 6), we notice that, while there is a general agreement between the two models, there are visible discrepancies at certain ages. These are more evident in very young individuals at age 1, and in older children from around age 8 onward, where the empirical model indicates a more noticeable peak for the *S*^−^ distribution.

Finally, the estimates for *δ* and 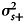 from the unified mechanistic and empirical models are comparable, with largely overlapping 95% confidence intervals (Table 1).

**Table 1.** Maximum likelihood estimates with associated 95% CIs (within brackets) for the unified mechanistic model (UFM) and empirical model (EM), fitted to the *Pf* AMA1 antibody data

Equation	Parameter	UFM	EM
Equations (9) and (11)	*l*_0_	−2.696 (−2.627, −2.397)	
	*l*_1_	0.246 (0.202, 0.264)	
Equation (10)	*γ*	−1.5 (−1.687, −1.291)	
	*r*	3.806 (3.122, 4.754)	
Equation (12)	*δ*	31.086 (27.637, 37.837)	28.348 (25.265, 34.197)
	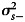	2.506 ⋅ 10^−3^ (2.169 ⋅ 10^−3^, 2.914 ⋅ 10^−3^)	1.895 ⋅ 10^−3^ (1.613 ⋅ 10^−3^, 2.288 ⋅ 10^−3^)
	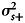	23.977 (15.783, 46.364)	36.063 (23.244, 70.104)
Equation (14)	*β*_1_		−3.141 (−3.191, −3.087)
	*β*_2_		0.052 (0.046, 0.058)
	*β*_3_		−0.021 (−0.032, −0.005)
Equation (15)	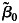		−3.031 (−3.194, −2.69)
	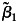		2.005 (1.915, 2.188)
	AIC	29 791.910	29 711.460

The Akaike Information Criterion (AIC) is also reported.

**Fig. 7.**
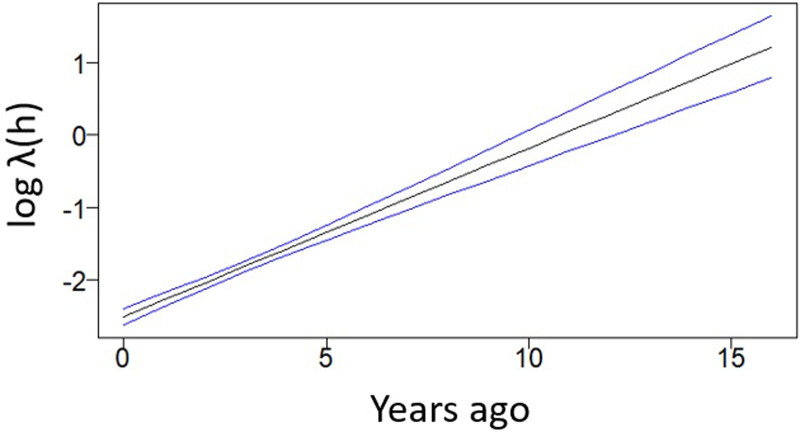
Changes in *λ* over historical time as derived from the unified mechanistic model fitted to *Pf* AMA1 antibody data. The blue lines indicate 95% CIs. ‘Years ago’ corresponds to (*a* − *h*) as described in (11).

With regards to *λ*(*h*), Figure 7 shows the estimated changes in this parameter in the 16 years before data collection. The results indicate a decrease in transmission in recent years.

Finally, based on the AIC, we note that the unified mechanistic model is larger, suggesting that inferences from the mechanistic model should be drawn with caution. This is because the mechanistic model may not provide an equally good description of the antibody distribution across all ages as shown by the discrepancies between the red and blue lines of Figure 6. However, because the differences between the models are not substantial, we believe that the unified mechanistic model does provide useful insights into time variations of the seroconversion and boosting rates, for which the empirical model does not provide any information.

## Discussion

We have introduced a unified mechanistic model which (1) avoids the dichotomisation of continuous antibody data and (2) provides a more flexible way for modelling antibody distributions while allowing for the joint estimation of seroconversion and boosting rates, namely *λ*(*a*) and *γ*(*a*), respectively.

The additional flexibility is obtained by modelling the age-dependency of antibody distributions and the temporal variations in *λ*(*a*) and *γ*(*a*) which are informed by RCM and AAM, respectively. The disadvantages of dichotomising continuous data into binary data, a common practice in the standard use of RCMs, are well established. Dichotomisation can lead to the loss of information which affects the ability to reliably recover regression relationships and the precision of parameter estimates [36–40]. The proposed unified modelling framework in this paper avoids this problem by making use of the full continuous antibody distribution.

As an alternative approach to the mechanistic framework, we have proposed the use of an empirical approach where the age dependency is informed by the data rather than by biological assumptions. The choice between the unified and empirical models may depend on the research context. The mechanistic approach allows for the estimation of *λ*(*a*) and *γ*(*a*) that may be of intrinsic scientific interests, whilst the empirical model does not provide any information on these. In our application, the empirical model provided a better fit to and, hence a better description of, the antibody distributions for different ages, although the discrepancies between the fitted antibody distributions of the empirical and unified models were minimal.

One of the main issues of the proposed unified modelling framework is that it requires a large amount of data in order to reliably estimate the model parameters. In cases where the separation between the seronegative and seropositive populations is weak, this may result in very uncertain estimates. For example, additional analysis of the antigen *Pf MSP*1_19_ showed limited evidence of a bi-modal distribution or age dependency in the mixture distribution, making the estimation of the proposed model unfeasible. More generally, mixture models may be difficult to estimate, especially in areas of high transmission where a great majority of the population is seropositive [3, 17]. Additionally, the seroreversion rate *ω* may also difficult to estimate in this scenario and, for this reason, is often fixed [4]. This is one of the main limitations in RCMs, which also applies to the unified mechanistic model. Generally, to alleviate the problem of over-parametrisation, further simplification of the model may be considered by, for example, assuming a constant *λ*(*a*). In such scenarios, however, we believe selection between models should also be guided by scientific, a not purely statistical, judgement, while also taking into consideration the levels uncertainty inherent to each model.

More complex functional forms for modelling time-changes in *λ*(*a*) and *γ*(*a*) than a log-linear regression, as used in this paper, could also be considered. For example, polynomials and smoothing splines would be a natural choice to increase the flexibility of the model. Alternatively, contextual knowledge on events that may have significantly impacted transmission in the past, such as interventions and malaria outbreak, may also be used to inform the modelling of *λ*(*a*) and *γ*(*a*). However, the increased flexibility comes at the cost of an increased model complexity which may make the model very difficult, if not impossible, to estimate.

## Conclusion

We have proposed a unified modelling framework for the analysis of malaria serology data which allows for the joint estimation of seroconversion and boosting rates. Our framework makes the best possible use of the data by avoiding the dichotomisation of the continuous antibody measurements, a common practice in the analysis of malaria serology data. More importantly, the unified framework allows to critically assess and evaluate assumptions on the heterogeneity of biological indicators of malaria transmission using a principled likelihood-based framework.

## Data Availability

R scripts for the implementation of the unified mechanistic and empirical models are available on request from the authors.

## Supplementary material

For supplementary material accompanying this paper visit http://dx.doi.org/10.1017/S0950268821000753. click here to view supplementary material
