# Supplementary material for: A unified and flexible modelling framework for the analysis of malaria serology data
Source: Epidemiol Infect. 2021 Apr 12;149:e99. doi: 10.1017/S0950268821000753 (PMC8161365; doi:10.1017/S0950268821000753)
Supplement: Supplementary file 1 [file S0950268821000753sup001.pdf]

Epidemiology and Infection.
A unified and flexible modelling framework for the analysis of malaria serology data.
Supplementary material.

Irene Kyomuhangi *[1] and Emanuele Giorgi[1]

[1]CHICAS, Lancaster Medical School, Lancaster University, UK
*i.kyomuhangi@lancaster.ac.uk

*Corresponding author

A Additional illustration of the mechanisms underlying the unified mechanistic model

A.1 Contribution of individuals of different ages to the estimation of transmission parameters λ and γ

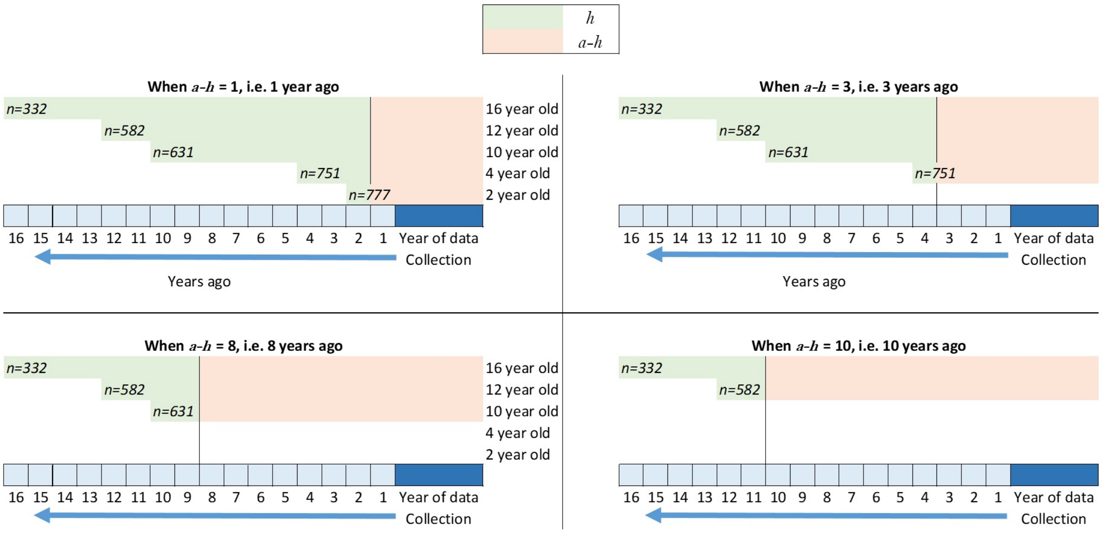

Figure. S 1: An illustration of how individuals of different ages contribute to estimation of λ and γ for *Pf* AMA1 through historical time. Data is taken from section 4. $a-h$ and h, as defined by equation 11, represent X-years ago, and the age of the individual X-years ago, respectively. For example in the top right panel, all individuals above 1 year will contribute to the estimation of γ one year ago, however in the bottom right panel, only individuals above 10 years will contribute to the estimation of γ 10 years ago. Note that individuals who contribute to the estimation of γ do so equally, regardless of how old they were at the time, i.e. regardless of the value of h. Also note that the further back in time we estimate γ, the fewer the number individuals, n, contribute to the estimate.

A.2 Model formulations for the unified mechanistic model

Time discretization of the RCM

Let $p(a)$ be the proportion of seropositive (S^+) individuals at age a. Given that individuals seroconvert from seronegative S^- to S^+ at rate $\lambda(a)$, and serorevert from S^+ to S^- at rate ω, the standard expression of the temporal dynamics in the RCM:

$$\frac{dp}{da} = \lambda(a)(1-p(a)) - \omega p(a)$$

We then approximate this as

$$\frac{dp}{da} \approx p(a) - p(a-1)$$

Therefore,

$$\begin{aligned}
p(a) - p(a-1) &= \lambda(a)(1-p(a)) - \omega(a) \\
p(a) - p(a-1) &= \lambda(a) - (\lambda(a)p(a)) - \omega p(a) \\
p(a) + (\lambda(a)p(a)) + \omega p(a) &= \lambda(a) + p(a-1) \\
p(a)(1+\omega+\lambda(a)) &= \lambda(a) + p(a-1) \\
p(a) &= \frac{1}{1+\omega+\lambda(a)}(\lambda(a) + p(a-1))
\end{aligned}$$

Assuming $\lambda(0) = 0$, it follows that $p(0) = 0$

It then follows that,

$$\begin{aligned}
p(1) &= \frac{1}{1+\omega+\lambda_1}(\lambda_1 + 0) \\
&= \frac{\lambda_1}{1+\omega+\lambda_1} \\
p(2) &= \frac{1}{1+\omega+\lambda_2}(\lambda_2 + \frac{\lambda_1}{1+\omega+\lambda_1}) \\
p(3) &= \frac{1}{1+\omega+\lambda_3}\left(\lambda_3 + \frac{1}{1+\omega+\lambda_2}\left(\lambda_2 + \frac{\lambda_1}{1+\omega+\lambda_1}\right)\right)
\end{aligned}$$

And more generally,

$$p(a) = \sum_{h=1}^{a} \frac{\lambda(h)}{\prod_{k=h}^{a}(1+\lambda(h-k)+\omega)}$$

Time discretization of the AAM

Let $\mu(a)$ be geometric mean antibody level of individuals at age a. Assuming anti-malaria antibodies of individuals are boosted at rate $\gamma(a)$ upon exposure, and decay at rate r in the absence of exposure, the standard expression of the temporal dynamics in the AAM:

$$\frac{d\mu}{da} = \gamma(a) - r\mu_a$$

We then apply the approximation

$$\frac{d\mu}{da} \approx \mu_a - \mu_{a-1}$$

which leads to

$$\mu(a) - \mu_{a-1} = \gamma(a) - r\mu_a$$

$$\mu_a = \frac{1}{1+r}\Big(\gamma(a) + \mu_{a-1}\Big)$$

Assuming $\gamma(0) = 0$, we have $\mu(0) = 0$

It then follows that,

$$\begin{aligned}
\mu(1) &= \frac{1}{1+r}\gamma \\
\mu(2) &= \frac{1}{1+r}\Big(\gamma + (\frac{1}{1+r}\gamma_1)\Big) \\
&= \frac{1}{1+r}\gamma_2 + \left(\frac{1}{1+r}\right)^2 \gamma_1 \\
\mu(3) &= \frac{1}{1+r}\left(\gamma_3 + \frac{1}{1+r}\gamma_2 + \left(\frac{1}{1+r}\right)^2 \gamma_1\right) \\
&= \frac{1}{1+r}\gamma_3 + \left(\frac{1}{1+r}\right)^2 \gamma_2 + \left(\frac{1}{1+r}\right)^3 \gamma_1\Big)
\end{aligned}$$

And more generally,

$$\mu(a) = \sum_{h=1}^{a} \gamma(h) \left(\frac{1}{1+r}\right)^{a-h+1}$$

B Implementation of the unified mechanistic model in Section 4

B.1 Akaike Information Criterion (AIC) comparisons for the implementation of the unified mechanistic model

Table. S 1: Preliminary analysis of Western Kenya data, comparing the AIC for the empirical model (EM) and unified mechanistic models (UFM) with time-varying λ & constant γ, constant λ & time-varying γ, and different values of ω.

Model	ω	AIC
EM	–	29711.460
UFM, constant λ, time-varying γ	Continuous	30166.680
UFM, time-varying λ, constant γ	Continuous	29801.920
	0.01	29791.910
	0.5	29800.680
	1	29799.920
